# Impact of agricultural subsidy on chemical fertilizer use: Empirical evidence of China’s Organic-Substitute-Chemical-Fertilizer policy based on double machine learning

**DOI:** 10.1371/journal.pone.0334751

**Published:** 2025-11-05

**Authors:** Lei Deng, Pengcheng Wan, Fangyu Ye, Jingjie Zhao

**Affiliations:** 1 College of Information, Beijing Wuzi University, Beijing, China; 2 Beijing Municipal Tax Service, State Taxation Administration, Beijing, China; PLOS ONE, UNITED KINGDOM OF GREAT BRITAIN AND NORTHERN IRELAND

## Abstract

The sustainable development of agriculture hinges on effective fertilizer management, and China’s experience with chemical fertilizer overuse highlights the challenges and opportunities in this domain. This study examines the impact of agricultural subsidy policy on chemical fertilizer use across 2319 counties from 2012 to 2022. By treating the “Action Plan for Organic-Substitute-Chemical-Fertilizer (OSCF) for Fruits, Vegetables and Tea” as a quasi-natural experiment, this study uses a Double Machine Learning model to analyze its effects on fertilizer use and the underlying mechanisms, considering technical and scale efficiency as mediating variables. The findings reveal that the OSCF policy has a significant negative effect on chemical fertilizer use, primarily by enhancing both technical and scale efficiency. This study further reveals regional heterogeneity in the policy’s effectiveness. The results imply that while the impact of the OSCF policy is generally beneficial, it is shaped by regional economic development, agricultural production structure and initial level of fertilizer use. This highlights the importance of tailored policy instruments to address regional disparities in agricultural practices and targeted strategies to maximize the OSCF policy’s impact on sustainable agricultural development. This study provides valuable insights for policymakers and farm managers to enhance the sustainability of agricultural practices.

## 1. Introduction

The sustainable development of agriculture is a global priority, and rational fertilizer management is crucial to achieving this goal. China, as the world’s largest fertilizer consumer, has witnessed a significant surge in chemical fertilizer usage over the past few decades. While this has played a crucial role in feeding the country’s massive population, it has also posed significant challenges in balancing agricultural productivity with environmental sustainability [1,2]. China consumes approximately 30% of the world’s fertilizers, an alarmingly high figure considering it possesses only 9% of the global arable land [3]. While chemical fertilizers have undoubtedly contributed to China’s food security [4,5], their overuse has led to diminishing returns in agricultural productivity [6]. According to the National Bureau of Statistics of China, chemical fertilizer consumption reached 59.8 million tons in 2016, a 5.8-fold increase from 1978, while agricultural production only grew by 83.7%. Moreover, excessive fertilizer use has resulted in severe environmental problems such as soil degradation [7], water eutrophication [8], and increased greenhouse gas emissions [9], all of which pose significant risks to human health [10,11].

Reducing chemical fertilizer overuse is crucial for ensuring food security, promoting environmental sustainability, and achieving long-term agricultural productivity [12–15]. To address this issue, China has implemented several policies aimed at reducing chemical fertilizer application. In 2015, the “Action Plan for Zero Growth of Chemical Fertilizer Use by 2020” was introduced, emphasizing the promotion of scientific fertilization techniques and improved fertilizer efficiency. Subsequently, in 2017, the “Action Plan for Organic-Substitute-Chemical-Fertilizer (OSCF) for Fruits, Vegetables and Tea” was launched, offering subsidies to incentivize the use of organic fertilizers and reduce reliance on chemical alternatives. While chemical fertilizer use has decreased in recent years, the average application rate per hectare in China remains significantly higher than the global average [1]. Therefore, reducing chemical fertilizer usage remains a challenging and ongoing task [16].

Numerous studies have explored effective strategies and driving factors behind farmers’ reduced chemical fertilizer application. Technological advancements have been identified as crucial in improving fertilizer use efficiency. For instance, precision farming technologies like soil testing and formula fertilization can enhance technical efficiency and reduce fertilizer use intensity without compromising crop yields [17,18]. Additionally, integrating organic and inorganic fertilizers, such as compost and slow-release fertilizers, can improve soil fertility and reduce greenhouse gas emissions [19,20]. Social factors and farmer characteristics also influence fertilizer application behavior. Studies have investigated the role of social capital, education level, and risk perception in shaping farmers’ adoption of sustainable fertilizer management practices [9,21]. Market factors also play a crucial role in promoting sustainable fertilizer management. For example, land transfer, cooperative membership, and access to multiple sales channels can reduce fertilizer use intensity [22–24]. Moreover, environmental awareness and information access are important factors influencing farmers’ fertilizer application decisions [25]. Studies have also explored the role of digital economy and information technology in enhancing farmers’ understanding of the environmental impacts of fertilizer use and promoting the adoption of sustainable practices [26,27].

Agricultural subsidies, serving as primary economic incentives, have emerged as a powerful policy instrument for promoting global green and sustainable agricultural practices [1,4]. Previous studies have examined the impact of agricultural subsidies, such as those for organic fertilizers and precision farming technologies, on chemical fertilizer use. Some studies suggest that agricultural subsidies can effectively reduce the excessive use of chemical fertilizers [1,16,28–31]. However, other evidence indicates that agricultural subsidies may instead lead to increased chemical fertilizer use [32,33]. The mixed findings from prior studies can be attributed to two main factors. Firstly, the focus of these studies differs regarding the specific agricultural subsidy policies examined. Studies suggesting that agricultural subsidies increase chemical fertilizer use often focus on subsidies aimed at enhancing agricultural output or improving farmer incomes. These subsidies often promote chemical fertilizer use and expansion of planting areas, thus encouraging increased fertilizer application. In contrast, studies with opposing findings tend to focus on green agricultural subsidies or new agricultural subsidies, which are typically directed towards green production behaviors like organic fertilizer use and soil testing. These subsidies reduce chemical fertilizer use through the substitution effect of agricultural production inputs. Secondly, agricultural production is influenced by various inputs, making the impact of agricultural subsidies multi-dimensional [34]. Subsidies not only directly incentivize the use of targeted agricultural inputs but also shape the allocation of all agricultural inputs, thereby affecting chemical fertilizer use [4]. To analyze the multi-dimensional effects of agricultural subsidies, some recent studies have explored the underlying mechanisms through which agricultural subsidies impact chemical fertilizer use. These mechanisms primarily involve the scale effect and technical effect. For instance, agricultural subsidies encourage farmers to expand arable land scale and adopt new technologies, influencing chemical fertilizer use through these pathways [35]. Other studies also indicate that agricultural subsidies can affect chemical fertilizer use by promoting the use of agricultural machinery and expanding planting areas [1,16].

While these studies have significantly contributed to understanding the effects of agricultural subsidies on fertilizer application, a definitive consensus regarding these effects has not yet been achieved. Additionally, the OSCF policy, introduced in 2017 with the aim of substituting chemical fertilizers with organic fertilizers, represents a significant nationwide initiative. This policy involves redirecting agricultural subsidies to support producers who commit to integrating organic fertilization methods into their agricultural practices. The policy reform aims to encourage the scaling up of eco-friendly farming operations and promote the utilization of advanced organic fertilization techniques, thereby enhancing the ecological sustainability and productivity of the agricultural sector. Despite the OSCF policy having been implemented in China for several years, research examining its impact is limited. For example, Deng and Zhao [4] investigate the impact of varying subsidies on chemical and organic fertilizer use using cross-sectional micro-data and discusses the optimal subsidy for the OSCF. Similarly, Yi et al. [36] analyze the role of farmers’ psychological cognition in the adoption of the OSCF, while Yi et al. [37] employ two cross-sectional micro-data sets, one before and one after the policy implementation, to evaluate the effect of the OSCF policy on the reduction of chemical fertilizer use. However, existing literatures on the OSCF policy exhibit several limitations, leading to a poor understanding of its impact. Firstly, as a nationwide subsidy policy, cross-sectional micro-data from specific regions cannot provide a comprehensive understanding of its impact on fertilizer use. Secondly, existing studies have not adequately addressed the issue of endogeneity caused by omitted variables. Thirdly, the heterogeneity of policy effects and the underlying mechanisms through which the OSCF policy influences fertilizer use have not been systematically examined in existing studies.

While existing studies on agricultural subsidies have advanced our understanding of their impacts, traditional econometric approaches such as difference-in-differences (DID) and instrumental variable methods often struggle with high-dimensional confounders and unobserved heterogeneity. For instance, evaluations of the EU’s Common Agricultural Policy (CAP) frequently rely on cross-sectional or panel data models that inadequately address input endogeneity and self-selection bias [38,39]. Studies on CAP subsidies for beef production in Ireland, France, and Germany highlight how partial decoupling reforms may conflate farmer self-selection with policy effects due to insufficient causal controls [40]. Similarly, analyses of U.S. Farm Bill programs like the Conservation Security Program reveal challenges in disentangling policy impacts from regional heterogeneity and path-dependent labor adjustments [41,42]. Besides, EU and U.S. subsidy models reveal persistent trade-offs between economic incentives and environmental outcomes, driven by fragmented implementation and conflicting policy goals [43,44]. These limitations underscore the need for robust causal inference frameworks that account for both technical and scale efficiency pathways, which are often neglected in traditional studies.

Notably, the Double Machine Learning (DML) approach has proven robust in addressing high-dimensional confounders and complex causal relationships across diverse policy domains. For example, Ghosh et al. [45] applied DML to clarify the causal link between excessive body weight and hypertension risk, mitigating bias from observational data amid complex interactions. In addition, Lu and Kwan [46] employed a neural network-based DML framework to explore how noise exposure affects negative emotions and health across contexts, highlighting its ability to model non-linear, context-dependent relationships. Beyond these, Knaus [47] applied DML to estimate the effects of musical practice on students’ skills, showcasing its utility in parsing nuanced relationships within educational contexts. These applications demonstrate DML’s efficacy in addressing high-dimensional confounders, mitigating bias, and uncovering causal mechanisms, supporting its use in studying the OSCF policy’s impact on fertilizer use amid diverse influences.

In summary, there are three main limitations in existing literatures: First, prior research on agricultural subsidies exhibits mixed findings. This inconsistency stems from a narrow focus on specific subsidy types or regional case studies, neglecting nationwide policy evaluations. Second, while mechanisms such as technical and scale efficiency are theorized to mediate policy impacts, empirical evidence remains sparse, particularly in disentangling these pathways using robust causal methods. Third, existing studies often overlook the heterogeneous impacts of subsidies across regions with varying economic structures, initial fertilizer use levels, and crop types—a critical oversight given China’s vast regional disparities. Against this backdrop, we address two key questions: 1) Does the OSCF policy effectively reduce chemical fertilizer use at a national scale, and how does this effect vary across regions? 2) Through what mechanisms does the OSCF policy achieve reductions?

To answer these questions, this study employs county-level data from 2012 to 2022 and use the double machine learning (DML) approach to empirically analyze the impact of the OSCF policy on chemical fertilizer use. This study makes several potential contributions to the existing literature on agricultural subsidy policies. Firstly, by utilizing nationwide county-level macro-data, this study provides a more comprehensive and systematic understanding of the OSCF policy, offering valuable insights for policy formulation. Secondly, this study pioneers the application of DML in the field of agricultural subsidy analysis, effectively mitigating the challenges associated with dimensionality and selection biases, thus enhancing the robustness and reliability of the empirical findings. Thirdly, this study explores the regional heterogeneity in the impact of the OSCF policy and the mechanisms through which it affects fertilizer application, providing a more nuanced understanding of the policy’s effectiveness.

The rest of the study are organized as follows. Section 2 outlines the policy background and formulates the research hypotheses. Section 3 is allocated to describing the methodological approach and data source. Section 4 provides a comprehensive analysis of the study’s results. Section 5 concludes the study’s results and research limitations.

## 2. Background and theoretical analysis

### 2.1. Policy background

China’s agricultural industry has witnessed a marked escalation in chemical fertilizer use over recent decades. In 2000, the total consumption of chemical fertilizers (in pure terms) reached 46.37 million tons, peaking at 60.23 million tons in 2015 (https://data.stats.gov.cn/). Although the use of chemical fertilizers has since declined to 58.59 million tons in 2016, it remains significantly higher than the global average [1]. This excessive reliance on chemical fertilizers poses a significant threat to environmental sustainability and human health, contributing to soil acidification, water eutrophication, and ecological deterioration [48].

To address this issue, China’s Ministry of Agriculture and Rural Affairs introduced the “Action Plan for Organic-Substitute-Chemical-Fertilizer (OSCF) for Fruits, Vegetables and Tea” in 2017. This policy recognizes the potential of organic fertilizers, such as animal manure and agricultural by-products, to improve soil health and reduce environmental contamination. It aims to replace chemical fertilizers with organic alternatives in the production of fruits, vegetables, and tea, emphasizing resource recycling, cost-effectiveness, quality enhancement, and the exploration of a modern agricultural development path that is efficient, safe, resource-conserving, and environmentally friendly. The number of pilot counties expanded from 100 in 2017 to 238 in 2021. The policy calls for a 15% reduction in chemical fertilizer use in core production areas and well-known brand production bases within the pilot counties, with the aim of achieving zero growth in overall fertilizer use. Additionally, the policy emphasizes the development of circular agriculture by promoting the use of organic fertilizers, aiming to increase their use by over 20% in core production areas. To achieve these targets, the Chinese government allocated one billion CNY from its central budget in 2018 to support the OSCF, primarily focusing on subsidies for commodity and compost organic fertilizer, integration of water and fertilizer (including equipment and water-soluble fertilizer), soil testing, fertilizer formula test and fertilizer effectiveness evaluation, and technical training.

### 2.2. Theoretical analysis and research hypothesis

**Fig 1:**
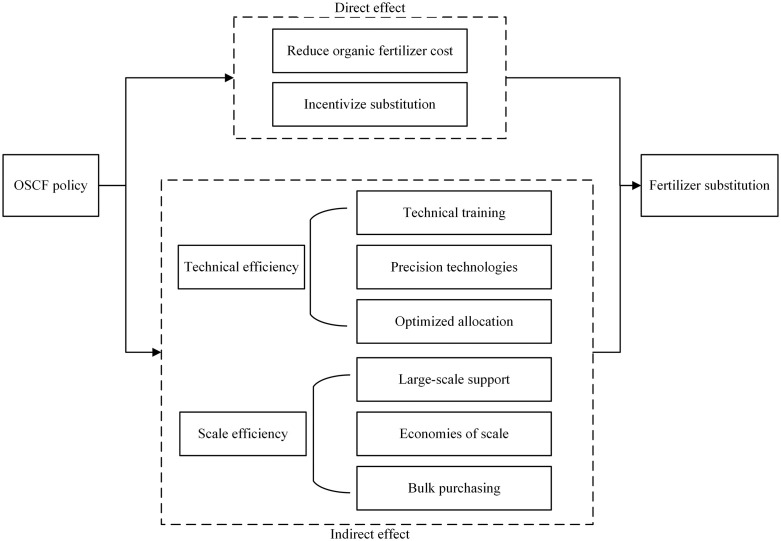
Influence mechanism of the OSCF policy on chemical fertilizer use.

Profit maximization is a well-established analytical framework for understanding farmers’ behaviors and evaluating the effectiveness of policy instruments [1]. For rational farmers, the influence of agricultural subsidies on farm production can be conceptualized as the strategic allocation of agricultural inputs within a set of constraints, with the ultimate goal of maximizing profit. Two primary constraints faced by farmers are liquidity constraints and the scarcity of arable land [49,50]. Liquidity constraints hinder farmers’ ability to invest in environmentally-friendly agricultural inputs and technologies [51]. Moreover, the significant barrier to the adoption of chemical fertilizer substitutes, such as organic fertilizer, is the high costs in terms of agricultural inputs expenditure, investment in equipment and labor demand. Consequently, there is a lack of economic incentive for farmers to use chemical fertilizer substitutes [4,16]. The OSCF policy, however, can alleviate these liquidity constraints. It directly reduces chemical fertilizer use by incentivizing farmers to adopt more organic fertilizers as substitutes. Additionally, it encourages farmers to adopt advanced techniques and transition towards more intensive and large-scale agricultural production practices, further reducing chemical fertilizer use. Fig 1 illustrates how the OSCF policy drives reductions in chemical fertilizer use.

Firstly, the high costs of organic fertilizers significantly dampen the incentive for farmers to transition away from chemical fertilizers [52]. The OSCF policy partially mitigates this barrier by directly reducing the cost of organic fertilizers [37,53], acting as a form of income subsidy [54]. This reduction improves farmers’ profit margins and expected farm income, enabling them to purchase and use more organic fertilizers to replace chemical fertilizers, thereby reducing chemical fertilizer use [55]. The subsidy for organic fertilizer, whether based on the quantity of organic fertilizer applied or the cultivated area, encourages increased organic fertilizer usage even when farmers are already using chemical fertilizers efficiently [4,56].

Secondly, Advanced equipment, production skills, and knowledge are often required for the application of environmentally-friendly chemical fertilizer substitutes and production techniques [57]. However, farmers in China typically face liquidity constraints, making it difficult to invest in human capital or advanced equipment [58]. The OSCF policy addresses this issue by supporting the adoption of environmentally-friendly techniques and providing technical training, reducing the risks associated with adopting new technologies and improving farmers’ ability to absorb new knowledge and technologies [16,59]. The adoption of new technologies promotes the rational allocation of agricultural inputs and improves agricultural productivity, thereby optimizing and reducing chemical fertilizer use [60]. These interventions align with Farrell’s efficiency framework [61], which posits that optimizing input use (e.g., reducing excess fertilizer) requires both knowledge diffusion and technology adoption. By lowering the cost of precision tools, the OSCF directly addresses allocative inefficiency—the gap between actual and optimal input use [62].

Thirdly, small-scale farms and the management practices of smallholders are significant barriers to mitigating the excessive chemical fertilizer use in China [1]. In contrast, large-scale farming has emerged as a promising avenue for ensuring food security while promoting sustainable agricultural development [63,64]. Facing the liquidity constraints, farmers operating small-scale farms often have to overuse chemical fertilizer to compensate for the lack of investment in labor and advanced technologies [16,65]. The OSCF policy primarily targets large farms, family farms, agricultural cooperatives, and agricultural enterprises, facilitating an enhancement in the scale and specialization of agricultural production. Additionally, the OSCF policy emphasizes the establishment of large-scale demonstration areas, contributing to the expansion of agricultural production scales in the pilot areas. Large-scale farms benefit from economies of scale, leading to more efficient resource allocation and reducing chemical fertilizer use [32]. Moreover, large-scale farmers often possess advanced management knowledge and skills, enabling them to improve their cultivation methods and achieve sustainable development and improved agricultural productivity, further reducing chemical fertilizer use [66–68]. In summary, the OSCF policy can reduce chemical fertilizer use by prompting farmers to achieve economies of scale, thereby improving the utilization efficiency of agricultural inputs and agricultural productivity. This reflects economies of scale theory [69], where larger operational scales reduce per-unit input costs (e.g., fertilizer) through mechanization and bulk purchasing.

Based on the above analysis, the hypothesises of this study are put forward:

**Hypothesis 1.** (H1): The OSCF policy can reduce chemical fertilizer use.
**Hypothesis 2.** (H2): The OSCF policy reduces chemical fertilizer use by improving agricultural technical efficiency.
**Hypothesis 3.** (H3): The OSCF policy reduces chemical fertilizer use by improving agricultural scale efficiency.

## 3. Research design

### 3.1. Variable setting

#### 3.1.1. Explained variables.

The OSCF policy mainly support the substitution chemical with organic fertilizer in the production of fruits, vegetables and tea. As we lack data on chemical fertilizer application for these crops, the total amount of chemical fertilizer per hectare in pure terms (*Y*) is used to measure the intensity of chemical fertilizer use. This measurement offers several distinct advantages. Firstly, Chinese farmers commonly use a variety of chemical fertilizers, including nitrogenous, phosphatic, potassic, and mixed fertilizers [70]. Given the diverse units used to measure these fertilizers, assessing chemical fertilizer use in terms of its pure content provides a more precise and standardized metric. Secondly, one of the key targets of OSCF policy is to reduce the application of chemical fertilizers of the pilot counties by more than 15%, and to influence the county-wide utilization of chemical fertilizers to achieve zero growth. Therefore, evaluating the impact of the OSCF policy using the total chemical fertilizer application across the entire county is deemed reasonable. This approach allows for a more comprehensive assessment of the policy’s effects, thereby offering more valuable insights for the formulation of other related policies. Thirdly, our measurement of the intensity of chemical fertilizer use is consistent with previous studies [16,71–73], ensuring research consistency and comparability.

#### 3.1.2. Explanatory variables.

This study examines the impact of the OSCF policy on 187 treatment counties that participated in the pilot program, compared to 2132 control counties that did not. The policy’s effect is assessed using a dummy variable (*OSCF*) indicating the year the pilot program was initiated in each county.

#### 3.1.3. Control variables.

This study uses the regularization algorithm of DML to handle high-dimensional control variables effectively. The selection of control variables draws upon existing literature [1,16,71,74–81] and considers the availability of county-level data. The study includes variables across several dimensions to capture the multifaceted factors influencing agricultural production and fertilizer use.

***Economic and Policy Dimension:*** Government fiscal spending (*GFS*) is expressed as proportion of government spending to GDP. Fiscal expenditures in healthcare are measured by hospital beds per 10,000 population (*HSH*) and medical staff per 10,000 population (*HSM*). Economic development level (*EDL*) gauged by the per capita GDP. Stringency of policy and technical training (*SPT*) is calculated by the number of committees per town. Urban-rural income distribution (*URD*) is measured by the ratio of rural per capita net income to urban per capita disposable income. Regional economic marketization level (*REM*), gauged by the ratio of the total retail sales of consumer goods to total population.

***Industry and Structural Dimension:*** Integrated mixed crop-livestock production (*MCL*) is expressed as the ratio of the agricultural value-added to husbandry value-added. Agricultural development level (*ADL*) is measured by the ratio of agricultural output value to total arable land area. Agricultural industry scale (*AIN*) is expressed in terms of the ratio of total arable land area to administrative area. Land scale operation (*LSO*) is expressed as the ratio of total arable land area to number of rural households. Industrial structure (*INS*), gauged by the proportion of primary industry value-added to GDP. Agricultural industry structure (*AIS*) is expressed as the ratio of grain crop sown area to total arable land area. Natural environment (*NE*) is expressed in terms of annual average precipitation. Value of agricultural production (*VAP*) is measured by the ratio of total agricultural output value to crop yield. Agricultural marketization level (*AML*), gauged by the ratio between agricultural industry employed population and administrative area. Agricultural output level (*AOL*) is expressed as the per capita crop yield.

***Population and Household Dimension:*** Off-farm employment (*OFE*) is measured by the average number of rural employees per household. Off-farm income (*OFI*) is expressed as off-farm employment multiplied by average monthly minimum wage. Household size (*HS*), gauged by the ratio of rural population to number of rural households. Farm income (*FI*) is measured by the net income of rural residents. Farm consumption (*FC*) is expressed in terms of per capita expenditure of rural residents. Agricultural inputs (*AGI*), represented by the rural power consumption. Anti-risk capacity (*ARC*) is measured by the per household savings balance. Urban-rural population structure (*SUR*), gauged by the ratio between rural population and total population.

***Science and Technology Development Dimension:*** Agricultural labor productivity (*ALP*) is expressed as the ratio between agricultural value-added and agricultural industry employed population. Agricultural mechanization (*AM*) is calculated by the ratio of total agricultural machinery power to total crop land area. Education Level (*EL*) is gauged by the number of students per 10,000 population. Internet development level (*IDL*) is expressed as the ratio of internet users to total population. Information transmission and sharing (*IST*) is measured by the ratio of telephone and mobile phone users to total population.

#### 3.1.4. Mechanism variables.

While the OSCF policy directly encourages the substitution of chemical with organic fertilizer, its indirect impact on reducing chemical fertilizer use is mediated through improved agricultural technical and scale efficiency. Previous studies have typically defined these indirect effects as technical and scale effects, and measured by agricultural machinery input and farmland scale, respectively [1,16]. However, the OSCF policy’s multifaceted influence on farmers’ practices and input choices necessitates a more comprehensive approach. Therefore, to comprehensively evaluate how the OSCF policy reduces fertilizer use by influencing farmland scale and technology adoption and referring to the existing literature [66], this study employs scale efficiency (*SE*) and technical efficiency (*TE*) to measure the scale and technical effects of the OSCF policy.

This study utilizes the Data Envelopment Analysis (DEA) model to evaluate agricultural efficiency. The output indicator is the agricultural value-added, adjusted for price factors. The input indicators encompass the total arable land area, chemical fertilizer use (pure), total power of agricultural machinery, the number of agricultural employees, the rural power consumption, and the annual average precipitation. The input-oriented Malmquist productivity index method, under the assumption of constant returns to scale, is applied to decompose the total factor productivity (TFP) into *TE* and *SE* (S1 File provides the details of the estimation process of TE and SE).

#### 3.1.5. Data sources.

This study evaluates the macro-level impact of the OSCF policy on chemical fertilizer use in China. The data are mainly sourced from China County Statistical Yearbook and local statistical yearbooks of each province and prefecture-city. Specifically, chemical fertilizer use, rural power consumption, expenditures of rural residents, monthly minimum wage, the net income of rural residents, and the employed population in agricultural industry are sourced from local statistical yearbooks from each prefecture-level city. Annual average precipitation is derived from the ERA5-Land dataset. Cultivation area and yield data for various crops are collected from county government websites. Missing data are provided through the China Economic and Social Big Data Research Platform on the official website (https://data.cnki.net/) as much as possible. The list of pilot counties selected in 2017 is obtained from the Ministry of Agriculture and Rural Affairs of China. However, subsequent batches of pilot counties are non-public. Therefore, data for these counties is collected manually through formal requests for public disclosure submitted to the agricultural departments of each province, municipality, and autonomous region.

The data spans from 2012 to 2022, which is based on the following reasons. Firstly, the 18th National Congress of the Communist Party of China in 2012 officially advocated for ecological civilization, emphasizing protection and harmony with nature and initiating significant environmental policy changes. Secondly, starting the analysis in 2012 ensures symmetry between the pre-treatment (2012–2017) and post-treatment (2017–2022) periods, facilitating a more straightforward comparison between the two periods and deepens our comprehension of the policy’s impact [82].

Data processing involves excluding provinces with unavailable information of polit counties (For example, the responses of Fujian, Shandong and Sichuan Province indicate the data are not public and unavailable), removing samples lacking necessary indicators, and supplementing missing data of remaining samples with corresponding county data from statistical yearbooks or interpolation. Specifically, interpolation for missing data is addressed through a three-tiered imputation protocol: firstly, for administrative or stable variables (e.g., institutional data with low volatility), missing values are filled using forward/backward filling to leverage temporal consistency; secondly, for time-trend variables (e.g., economic indicators), missing values are predicted using spatial-temporal interpolation based on adjacent counties’ data and historical trends; thirdly, remaining gaps are addressed via adaptive K-nearest neighbors imputation (Euclidean distance), which weights neighbors by probability density to preserve data distribution [83]. Then to mitigate potential biases arising from unit differences and skewness issues, particularly to address heteroscedasticity [84,85], all variables except dummy variables are normalized using z-score normalization. Descriptive statistics for the main variables are presented in Table 1.

**Table 1:** Descriptive statistics of main variables.

Variables	Mean	Min	Max	Std
*Y*	0.6113	0.0778	186.1051	3.7965
*OSCF*	0.0259	0.0000	1.0000	0.1588
**Economic and Policy**				
*GFS*	0.2804	0.0318	3.9407	0.2464
*HSH*	62.3199	1.2816	2904.7895	105.6009
*HSM*	66.5028	0.8065	3383.1053	105.5971
*EDL*	38870.3448	3854.4545	204629.6651	25909.3070
*SPT*	19.2232	1.1667	131.8000	13.9626
*URD*	0.7871	0.0267	0.8993	0.2180
*REM*	13463.6814	513.1515	102583.8235	10395.0835
**Industry and Structural**				
*MCL*	3.1816	0.0413	150.2857	9.0599
*ADL*	3.4597	0.0804	378.3308	11.4860
*AIN*	0.0385	0.0001	0.2664	0.1065
*LSO*	0.7125	0.0016	16.2233	0.7915
*INS*	0.1336	0.0030	0.5157	0.1068
*AIS*	0.0593	0.0000	0.5779	0.0437
*NE*	0.0031	0.0001	0.0104	0.0015
*VAP*	2.0532	0.0338	18.1655	3.3983
*AML*	77.4020	0.0348	628.3469	70.7374
*AOL*	0.6531	0.0079	36.8452	0.6079
**Population and Household**				
*OFE*	2.0578	0.3230	15.2824	0.7070
*OFI*	2768.5257	454.4639	21576.4778	1094.7781
*HS*	2.4352	1.0523	9.4964	2.8288
*FI*	14415.5640	2081.0000	62402.6655	6278.8996
*FC*	9324.4174	1159.0000	49202.1576	4537.6436
*AGI*	1.0218	0.4468	670.3361	10.8142
*ARC*	8.8981	0.3806	515.9096	7.8590
*SUR*	0.1620	0.0146	0.6437	0.1321
**Science and Technology**				
*ALP*	3.0758	0.3042	95.6681	2.9883
*AM*	8.8318	0.0287	87.5167	8.5901
*EL*	1215.7974	84.6800	3997.3333	506.3607
*IDL*	0.2314	0.0283	0.5581	0.0954
*IST*	0.8710	0.3182	0.9206	0.4745
**Mechanism variables**				
*TE*	1.0717	0.1228	5.0739	0.2991
*SE*	1.0522	0.1425	2.4537	0.1689

### 3.2. Double Machine Learning model construction

This study employs the DML approach to precisely evaluate the causal impact of the agricultural subsidy on chemical fertilizer use. The OSCF policy serves as a case of an exogenous policy shock, providing a unique opportunity for causal inference. Traditional causal inference methods, such as DID, synthetic control methods (SCM), and regression discontinuity design (RDD), often face limitations in addressing high-dimensional data, non-linear relationships, and endogeneity caused by omitted variables [86,87]. For instance, DID requires strict parallel trends assumptions and struggles with heterogeneous treatment effects, while SCM is sensitive to extreme values in the treatment group [88]. In contrast, DML excels in handling complex datasets, overcoming the limitations of traditional multiple linear regression models in dealing with non-linear relationships and the ‘curse of dimensionality’ [89,90]. Its robust framework effectively addresses challenges such as multicollinearity and high-dimensional variables, enhancing estimation accuracy [91,92].

Chemical fertilizer use is influenced by a multitude of factors, including economic, political, and social factors [93]. To optimize policy effect estimation, it is critical to control for high-dimensional confounders. Traditional regression models face the “curse of dimensionality” when incorporating numerous control variables, leading to biased estimates due to multicollinearity or oversimplified linear assumptions [94]. DML mitigates these issues by employing regularization algorithms to automatically filter and select relevant variables from high-dimensional data, thereby reducing bias and improving predictive accuracy [87,95]. For example, DML decomposes the estimation into two stages: first, predicting residuals of the outcome and treatment variables using machine learning models (e.g., random forests or Lasso), and second, estimating the causal effect via orthogonalized residuals [95]. This approach avoids regularization bias and model misspecification, which are common pitfalls in traditional methods [47,96].

Furthermore, the relationship between agricultural subsidies and fertilizer use is inherently complex and potentially non-linear. Linear regression models impose restrictive parametric assumptions, failing to capture interactions or threshold effects [97]. DML’s flexibility allows it to model both linear and non-linear relationships through machine learning algorithms like random forests or neural networks, which adaptively learn data patterns without pre-specified functional forms [98–100]. For instance, the OSCF policy’s impact may vary with regional economic development or initial fertilizer use levels-heterogeneities that DML can uncover through conditional average treatment effect estimation [96].

Endogeneity arising from unobserved confounders or reverse causality is another critical concern. Traditional instrumental variable approaches require valid instruments, which are often unavailable or weakly identified. DML addresses this by leveraging high-dimensional controls to approximate the confounding structure, thereby reducing omitted variable bias [87,95]. For example, our model incorporates 30 control variables across economic, structural, and technological dimensions, which traditional methods struggle to accommodate without overfitting.

In summary, DML offers three key advantages for this study: firstly, Robust handling of high-dimensional data through regularization and variable selection, mitigating multicollinearity and dimensionality challenges. Secondly, flexibility in modeling non-linear relationships via machine learning algorithms, avoiding restrictive parametric assumptions. Thirdly, reduced endogeneity bias by orthogonalizing treatment effects against confounders, enhancing causal identification. These strengths align with the complexities of evaluating the OSCF policy, making DML a superior choice over traditional methods for this research.

Therefore, the study employs DML to assess the policy’s effect. The impact of the OSCF policy is evaluated through a two-stage process. In the first stage, the conditional estimations of residuals are decomposed into two separate prediction tasks: one for the outcome and another for the treatment. In the second stage, the average treatment effect is estimated using a final predictive model, incorporating the residuals from the outcome and treatment models [95]. Additionally, to account for the potentially complex and non-linear relationship between the OSCF policy and chemical fertilizer use, as well as to address heterogeneity in model estimation and mitigate the subjectivity inherent in the widely used partial linear models [86], this study uses a more generalized interactive model:


$$Y_{it}=g\left({OSCF}_{it},X_{it}\right)+U_{it},\;E\left(U_{it}|{OSCF}_{it},X_{it}\right)=\text{0}$$ (1)

$${OSCF}_{it}=m\left(X_{it}\right)+V_{it},\;E\left(V_{it}|X_{it}\right)=\text{0}$$ (2)

In this model, *i* represents the county and *t* denotes the year. *Y* is the explanatory variable related to the intensity of chemical fertilizer use. The dummy variable *OSCF* denotes the treatment variable associated with the OSCF policy, taking a value of 1 after the pilot program implementation and 0 otherwise. *X* is a high-dimensional vector of control variables. And *U*, *V* represent the error terms of the primary and auxiliary regressions, respectively.

## 4. Results and discussion

### 4.1. Impact of OSCF policy on chemical fertilizer use

This study employs the DML approach to assess the impact of the OSCF policy on chemical fertilizer use. The sample is divided using a 1:4 ratio, and random forest models are employed for primary and auxiliary regression predictions, and Lasso and neural network algorithms are used in the robustness tests. Our choice of machine learning algorithms for the DML framework is guided by their complementary strengths in addressing the specific challenges of our dataset and research objectives. Alternatives like support vector regression may be computationally prohibitive for high-dimensional data and requires intensive kernel tuning, limiting reproducibility [101]. And the sequential tree-building of gradient boosting machines increases overfitting risks and computational costs without proportional gains in causal inference accuracy [102]. Given our focus on causal inference rather than pure prediction, random forest’s parallelized training and inherent regularization offered a more balanced tradeoff between accuracy and efficiency. Moreover, random forest excels at capturing complex, non-linear relationships and interactions among variables without requiring explicit specification [103]. This aligns with our need to model intricate socio-economic and agricultural dynamics in a high-dimensional setting. Besides, by aggregating predictions from multiple decorrelated trees, random forest mitigates overfitting while maintaining computational efficiency, making it suitable for large-scale county-level data. Additionally, random forest provides intuitive measures of variable importance, aiding in the interpretation of key drivers (e.g., economic development, initial fertilizer use) influencing policy effects [90,95,101]. On the other hand, the Lasso can efficiently select relevant covariates via L1 regularization, reducing multicollinearity and model complexity [91], and neural networks excels at modeling hierarchical and complex patterns in large datasets, ensuring scalability for 21,659 observations [98].

To ensure the robustness of our machine learning models, we rigorously optimize hyperparameters such as the number of trees, tree depth, and minimum leaf size in the random forest algorithm. Based on a preliminary analysis, we employ a stringent cross-validation to select hyperparameters that minimized out-of-sample prediction error while maintaining computational efficiency. For example, the number of trees was set to 100 to balance prediction stability and computational cost. This aligns with best practices in machine learning literature [103] and are validated through sensitivity analyses, confirming that results remained consistent across alternative hyperparameter configurations. The results are illustrated in Table 2.

**Table 2:** Benchmark regression results.

Variables	(1)	(2)	(3)	(4)	(5)
*OSCF*	−0.1206*** (0.0541)	−0.1024*** (0.0316)	−0.0982*** (0.0323)	−0.0855*** (0.0321)	−0.0681*** (0.0320)
Control variable linear term	No	No	Yes	Yes	Yes
Control variable quadratic term	No	No	No	Yes	Yes
County fixed effects	No	Yes	No	No	Yes
Time fixed effects	No	Yes	No	No	Yes
Observations	25509	25509	25509	25509	25509

Column (1) of Table 2 reveals a significantly negative impact of the OSCF policy on chemical fertilizer use at the 1% significance level. When controlling for county and time fixed effects in column (2), the results consistently show a negative and significant regression coefficient, with negligible changes in the coefficient value, thereby confirming the robustness of the findings. The model is further extended in columns (3) and (4) by incorporating primary and quadratic terms for control variables, respectively. In column (5), the model accounts for both county-fixed effects and time-fixed effects, as well as primary and quadratic terms of control variables, showing an increase in the regression coefficients for chemical fertilizer use associated with the OSCF policy. Nonetheless, the policy continues to have a significant negative effect on chemical fertilizer use, thereby supporting Hypothesis H1.

The findings are consistent with previous research that views agricultural subsidies as a potent policy instrument for promoting sustainable agricultural development [1,16]. As previously analyzed, the reduction in chemical fertilizer use can be attributed to three factors influenced by the OSCF policy. Firstly, the subsidy for organic fertilizer narrows the price gap with chemical fertilizers, encouraging farmers to adopt alternative organic fertilizers [4]. Secondly, the OSCF policy enhances farmers’ ability to absorb new knowledge and promotes the adoption of environmentally-friendly technologies [104], which in turn improves agricultural productivity and decreases overall chemical fertilizer consumption [60]. Thirdly, the policy’s linkage with large-scale farms incentivizes farmers to adopt large-scale and intensive cultivation modes, which typically operate on economies of scale, thereby improving agricultural productivity and reducing chemical fertilizer use [66]. The indirect effects of the OSCF policy on chemical fertilizer use through the improvement of agricultural productivity from technical and scale perspectives are further substantiated in the section dedicated to mediating effect analysis.

### 4.2. Robustness tests

This section aims to validate the robustness of the findings presented in the benchmark model. Several alternative methods are employed to assess the stability of the results across different analytical approaches and sample configurations. The robustness tests are conducted based on the benchmark model of column (5) in Table 2, and the results are presented in Tables 3 and 4. The robustness tests conducted using various methods consistently support the findings of the benchmark model. The OSCF policy is found to have a significant negative impact on chemical fertilizer use, and this effect is robust across different analytical approaches, sample configurations, and the presence of other concurrent policies.

**Table 3:** The results of the robustness test.

Variables	Winsorize	Lag effect	Split ratio set 3:7	Split ratio set 1:2	Neural network algorithm	Lasso algorithm	Adjusting research sample
*OSCF*	−0.0658*** (0.0315)	−0.0564*** (0.0335)	−0.0644*** (0.0404)	−0.0698*** (0.0323)	−0.0528*** (0.0396)	−0.0410*** (0.0229)	−0.0613*** (0.0424)
Control variable linear term	Yes	Yes	Yes	Yes	Yes	Yes	Yes
Control variable quadratic term	Yes	Yes	Yes	Yes	Yes	Yes	Yes
County fixed effects	Yes	Yes	Yes	Yes	Yes	Yes	Yes
Time fixed effects	Yes	Yes	Yes	Yes	Yes	Yes	Yes
Observations	25509	23190	25509	25509	25509	25509	21439

**Table 4:** Results after excluding the effect of other policies on chemical fertilizer use.

Variables	(1)	(2)	(3)
*OSCF*	−0.0577*** (0.0279)	−0.0326*** (0.0214)	−0.0288*** (0.0255)
ECD	−0.0098* (0.0101)		−0.0058** (0.0119)
NDR		−0.0293*** (0.0302)	−0.0228** (0.0310)
Control variable linear term	Yes	Yes	Yes
Control variable quadratic term	Yes	Yes	Yes
County fixed effects	Yes	Yes	Yes
Time fixed effects	Yes	Yes	Yes
Observations	25509	25509	25509

#### 4.2.1. Winsorization.

To mitigate the potential influence of extreme values on the estimation results, all variables in the benchmark model, with the exception of the treatment variable, are subjected to winsorization at the 1% and 99% quantile levels. This procedure replaces values below the 1% quantile with the 1% quantile value and values above the 99% quantile with the 99% quantile value. The subsequent regression analysis is conducted using the winsorized data. The results demonstrate a high degree of consistency with the benchmark model, indicating that the observed effects of the OSCF policy on chemical fertilizer use remain robust even in the presence of potential outliers.

#### 4.2.2. Lag effect.

Considering the possibility of a time lag in the effects of the OSCF policy, the dependent variable is replaced with one-period lagged chemical fertilizer use. This lagged variable is then used to assess the impact of the OSCF policy. The results reveal a significant negative effect of the OSCF policy on lagged chemical fertilizer use, suggesting that the observed reduction in chemical fertilizer use is not a temporary phenomenon but rather a persistent effect of the policy.

#### 4.2.3. Model resetting.

To address potential bias arising from the specific configuration of the DML model, several modifications are implemented. Firstly, the sample split ratio is altered from 1:4 to 3:7 and 1:2 to investigate the potential impact of the split ratio on the results. Secondly, the random forest algorithm is replaced with the Lasso and neural network algorithms to explore the influence of different machine learning techniques. Despite the variations in the estimated policy effects across different model configurations, the results consistently show a significant negative impact of the OSCF policy on chemical fertilizer use, demonstrating the robustness of the benchmark model findings.

#### 4.2.4. Sample adjustment.

Given the significant disparities in agricultural development across different regions in China, including all counties in the regression analysis may lead to biased estimates [105]. Therefore, three municipalities with underdeveloped agricultural industry (Beijing, Tianjin, and Shanghai) and three provinces with more advanced agricultural base (Henan, Jiangsu, and Heilongjiang) are excluded from the sample. The remaining counties are then used to re-estimate the regression model. The estimated coefficient of the OSCF policy remains significantly negative at the 1% level, thereby reaffirming the robustness of the benchmark regression results.

#### 4.2.5. Exclusion of parallel policies.

To accurately assess the impact of the OSCF policy on chemical fertilizer use, it is crucial to account for the potential influence of other concurrent policies. Two policies that may have overlapping effects on chemical fertilizer use during the research period are considered: the Ecological Civilization Demonstration Area Policy (ECD) and the National Digital Rural Pilot Policy (NDR). Firstly, the ECD, initiated in 2013, aims to promote sustainable regional development and includes regulations addressing agricultural non-point source pollution control [106]. This policy has the potential to influence chemical fertilizer use within the research period. Secondly, the NDR, proposed in 2020, promotes the development of digital economy and digital technology in rural areas, which may also impact chemical fertilizer use [75,107]. Both policies are included in the regression model as dummy variables indicating whether a county is part of the pilot area. The results show that the estimated coefficient of the OSCF policy remains significantly negative at the 1% level, even after controlling for the effects of these parallel policies. This indicates that the impacts of the OSCF policy on chemical fertilizer, although exaggerated, do not undermine the accuracy of the results.

#### 4.2.6. Falsification test.

To further validate the robustness of our findings and address potential endogeneity, we conduct falsification tests in addition to the original robustness checks. These tests aim to verify whether the observed policy effects are spurious or driven by unobserved confounders. The results are presented in Table 5. we first construct a placebo OSCF policy by randomly assigning fictitious treatment years to counties in the control group. Using the same DML framework, we estimated the ‘effect’ of this placebo policy on chemical fertilizer use. Results show no statistically significant impact, confirming that the true OSCF policy’s effect is not attributable to temporal trends or coincidental shocks. Then, we replace the actual treatment group (OSCF pilot counties) with a randomly selected subset of control counties and re-ran the analysis. The estimated coefficient for the fictitious treatment group is negligible and statistically insignificant, reinforcing that the true treatment effect arises from the OSCF policy rather than unobserved county-level characteristics.

**Table 5:** The results of the falsification test.

Variables	Placebo policy	Fictitious Treatment Group
*OSCF*	0.0319 (0.0130)	−0.0012 (0.0162)
Control variable linear term	Yes	Yes
Control variable quadratic term	Yes	Yes
County fixed effects	Yes	Yes
Time fixed effects	Yes	Yes

#### 4.2.7. Parallel trend test.

**Fig 2:**
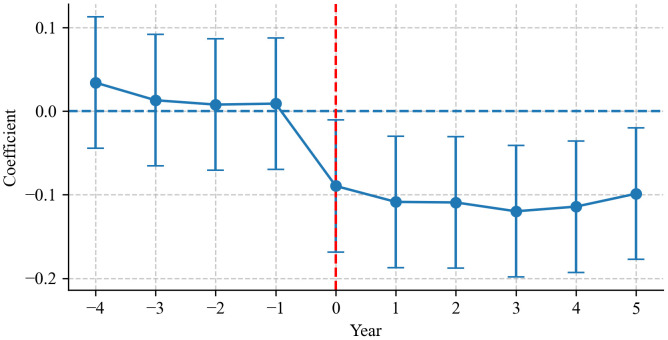
Parallel trend test.

A fundamental prerequisite for ensuring the validity of policy evaluation is that the treatment and control groups should follow parallel trends in chemical fertilizer use prior to the implementation of the OSCF policy. To verify this critical assumption, we employ an event study methodology. As illustrated in Fig 2, the results demonstrate that during the pre-policy period (2012–2016), there are no statistically significant differences in chemical fertilizer application between the pilot counties and others. This indicates that the two groups exhibit comparable evolving patterns in chemical fertilizer use before the policy intervention, thereby satisfying the parallel trend assumption. In contrast, after the implementation of the OSCF policy, a notable and statistically significant divergence in the chemical fertilizer use emerges between the two groups, which aligns with the expected policy effects. These findings lay a solid foundation for the reliability of the subsequent DML estimates, confirming that the observed differences in chemical fertilizer use post-policy can be reasonably attributed to the OSCF policy rather than pre-existing systematic disparities between the groups.

### 4.3. Heterogeneity analysis

#### 4.3.1. Developed and underdeveloped counties.

The economic disparity among different regions of China results in varying impacts of the OSCF policy on chemical fertilizer use across counties. Many scholars argue that environmental policy instruments may be more effective in developed areas due to higher investment in pollution control and a greater priority on environmental protection over economic growth [106,108,109]. To explore this, we categorize the sample counties into developed and underdeveloped regions by the median of GDP per capita and conduct separate regression analyses for each region. The results presented in Table 6 reveal that the OSCF policy significantly reduces chemical fertilizer use in developed areas, whereas this effect is minimal in underdeveloped regions. This aligns with previous studies, which demonstrate that the environmental policy instruments may be more effective in developed regions in China [110,111]. This suggests that the OSCF policy has a more pronounced impact on reducing chemical fertilizer use in developed areas, implying the OSCF policy may be more effective in these areas.

**Table 6:** Heterogeneity analysis results.

Variables	Economic development level		Initial chemical fertilizer use		Agricultural production structure	
	Developed areas	Underdeveloped areas	High chemical fertilizer use areas	Low chemical fertilizer use areas	Main grain production areas	Non-main grain production areas
*OSCF*	−0.158*** (0.0363)	−0.021** (0.0309)	−0.126*** (0.0331)	−0.046*** (0.0344)	−0.032 (0.0326)	−0.147*** (0.0317)
Control variable linear term	Yes	Yes	Yes	Yes	Yes	Yes
Control variable quadratic term	Yes	Yes	Yes	Yes	Yes	Yes
County fixed effects	Yes	Yes	Yes	Yes	Yes	Yes
Time fixed effects	Yes	Yes	Yes	Yes	Yes	Yes

This may be explained by the following reasons: Firstly, infrastructure and market access. Developed counties possess better rural infrastructure (e.g., organic fertilizer distribution networks, digital extension services), enabling efficient adoption of organic substitutes. In contrast, underdeveloped regions face logistical bottlenecks, raising transaction costs for organic inputs. [112]. Secondly, farmer capacity. Developed regions often have a higher economic base, which means that farmers can afford to invest in better agricultural inputs. Moreover, farmers in developed regions exhibit higher environmental awareness and technical literacy, facilitating the uptake of precision farming techniques promoted by the OSCF policy [113]. Thirdly, policy enforcement. Local governments in developed counties prioritize environmental goals over short-term economic gains, rigorously implementing OSCF targets [111].

To address these disparities, Tailored interventions are critical. For underdeveloped regions, complementary investments in rural infrastructure (e.g., organic fertilizer storage facilities) and farmer training programs are needed to amplify OSCF’s impact.

#### 4.3.2. High and low initial chemical fertilizer use counties.

The initial level of chemical fertilizer use may influence the effectiveness of the OSCF policy. Regions with higher pre-policy fertilizer use might face stricter environmental regulations and greater scrutiny from higher-level authorities, leading to increased policy implementation efforts and potentially enhancing its effectiveness [82,114,115]. Conversely, regions with lower initial fertilizer use might prioritize economic development and incentivize increased fertilizer application for yield enhancement, potentially diminishing the policy’s impact. To explore these potential differences, we divide the sample into high and low initial fertilizer use groups based on the median pre-policy average and conduct separate regression analyses.

Table 6 reveals that the impact of the OSCF policy on chemical fertilizer use varies across regions with different initial fertilizer use levels. The policy significantly reduces chemical fertilizer use in regions with high initial fertilizer use. As previously analyzed, this is possibly attributed to changes in farming practices or the economic incentives provided by the policy. In regions with low initial use, while the policy still leads to a decrease in fertilizer application, the effect is less pronounced. These findings are consistent with theoretical expectations and previous studies, which suggest that regions with higher initial pollution levels are more responsive to environmental policy interventions [106,110]. These results may be attributed to the following: (1) Regulatory pressure. High-use counties face stricter environmental oversight from central authorities, compelling local governments to prioritize fertilizer reduction [114,116]; (2) Marginal returns. Farmers in high-use areas experience diminishing returns from excessive fertilization, making them more responsive to subsidy-driven transitions [35]; (3) Behavioral lock-in. Low-use counties, often dominated by subsistence farming, prioritize yield stability over environmental benefits, resisting input substitution [51].

The heterogeneity in the OSCF policy’s impact on chemical fertilizer use, based on initial fertilizer use levels, highlights the need for tailored agricultural subsidy policies. Policymakers should consider regional variations in fertilizer use when designing these policies. High-use regions should receive performance-based subsidies to sustain reductions, while low-use areas require awareness campaigns highlighting long-term soil health benefits.

#### 4.3.3. Main and non-main grain production counties.

The OSCF policy primarily aims at subsidizing the substitution of chemical with organic fertilizer in the production of fruits, vegetables, and tea. However, considering the significant differences in agricultural input use, particularly fertilizer application, between grain and cash crops [50], it is imperative to investigate the varying impacts of the OSCF policy on chemical fertilizer use across main and non-main grain production regions. Therefore, we divide the sample counties into main and non-main grain production groups based on the median of the ratio between grain crop sown area and the total arable land area, and conduct separate regression analyses for each group. The results from these subgroup regressions, presented in Table 6, reveal that the OSCF policy significantly reduces chemical fertilizer use in non-main grain production regions but not in main grain production regions. These findings align with previous studies examining the effects of subsidy policies on agricultural input use [1,117].

The differential impact of the OSCF policy on chemical fertilizer use between grain and cash crop regions can be attributed to the following factors. Firstly, policy conflicts. Grain-producing counties are subject to parallel policies (e.g., grain security subsidies) that incentivize chemical fertilizer use to maximize yields. For example, to ensure food security, the Chinese government designated 13 main grain production regions in 2003 and implemented a comprehensive set of support policies, including financial incentives, input support, and technical support. These parallel policies may mitigate the impact of the OSCF policy on chemical fertilizer use in these regions [54]. Secondly, profit margins. Cash crops (e.g., fruits, tea) in non-grain regions have higher profit margins, allowing farmers to absorb the risks of organic transition. On the contrary, the grain storage and reserve policy (constituting a comprehensive set of measures implemented by the state to safeguard food security, maintain stability in the grain market, and uphold the interests of farmers), together with the lower market value of grain crops, may make grain crop farmers less responsive to changes in agricultural subsidy compared to cash crop farmers [118]. Thirdly, market integration: Non-grain regions are more integrated into premium markets (e.g., organic certification), creating demand for sustainable practices [31]. These findings underscore the importance of tailoring agricultural subsidy policies to the specific characteristics and needs of different regions and crop types. Policymakers should decouple grain security subsidies from chemical fertilizer use and introduce crop-specific OSCF incentives (e.g., higher subsidies for tea/fruit farmers).

### 4.4. Mediation analysis

Mediation analysis is a valuable tool for understanding the pathways through which an independent variable influences a dependent variable [119]. The theoretical analysis suggests that the OSCF policy’s impact on reducing chemical fertilizer use is primarily mediated by improvements in technical and scale efficiency. This section explores these underlying mechanisms using the technical efficiency (TE) and scale efficiency (SE) as mediating variables. The causal mediation analysis of DML is used to analyze these mechanisms based on the Lasso algorithm [100].

The first row of Table 7 presents the results of the mediation analysis focusing on the role of TE. The analysis reveals a significant negative direct and indirect effect of the OSCF policy on chemical fertilizer use in both the treatment and control groups, supporting Hypothesis H2. The OSCF policy facilitates investment in advanced agricultural technologies and practices by addressing liquidity constraints, enabling more precise fertilizer application and reducing chemical fertilizer use. Furthermore, training programs and knowledge transfer initiatives enhance farmers’ understanding of efficient fertilizer management, leading to more informed decision-making and reduced chemical fertilizer use. Additionally, the policy encourages the development of new and improved fertilizer technologies, offering more efficient alternatives to traditional fertilizers.

**Table 7:** Mechanism test results.

Variables	Total effect	Direct effect		Indirect effect	
		Treatment group	Control group	Treatment group	Control group
*TE*	−0.0332*** (0.0324)	−0.0308*** (0.0317)	−0.0219** (0.0267)	−0.0113** (0.0256)	−0.0024*** (0.0268)
*SE*	−0.0236*** (0.0495)	−0.0179*** (0.0524)	−0.0152*** (0.0503)	−0.0084*** (0.0317)	−0.0057* (0.0111)

The second row of Table 7 explores the mediating role of SE. The analysis demonstrates a significant negative indirect effect of the OSCF policy on chemical fertilizer use in both the treatment and control groups. After accounting for the SE pathway, the direct effects remain significantly negative, supporting Hypothesis H3. The OSCF policy promotes the consolidation of operations and adoption of larger-scale production methods. Larger-scale farms benefit from economies of scale, leading to more efficient resource allocation and enabling investment in better infrastructure and sophisticated management practices, including efficient fertilizer management. These factors contribute to reduced chemical fertilizer use in large-scale farming operations.

### 4.5. Feature importance

To enhance the interpretability of our machine learning-based analysis, we further estimated the importance of different features using random forest feature importance scores (based on mean decrease in Gini impurity). This allows us to clarify which pre-specified control variables most strongly shape the relationships between the OSCF policy and chemical fertilizer use.

**Fig 3:**
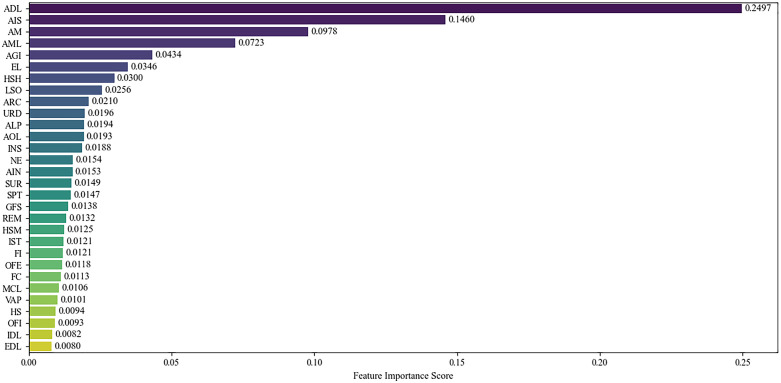
Feature importance.

The importance scores reveal a clear hierarchy aligned with agricultural production principles and policy implementation realities (Fig 3). Agricultural development level (ADL) emerges as the most influential factor, reflecting its foundational role in shaping resource allocation efficiency—higher ADL corresponds to more standardized production management and technological adoption, directly reducing fertilizer waste through improved technical efficiency [54]. Agricultural industry structure (AIS) ranks second, validating our heterogeneity findings that non-grain crop regions (focused on fruits, vegetables, and tea) exhibit stronger policy responsiveness due to the targeted design of OSCF measures [37].

Agricultural mechanization (AM) and agricultural marketization level (AML) highlight the importance of technical progress and market mechanisms: mechanization enhances precision in fertilizer application [16], while higher marketization facilitates coordinated promotion of organic fertilizer substitutes through supply chain networks [23]. Education level (EL) further supports the role of human capital in adopting scientific fertilization practices [113]. Land scale operation (LSO) aligns with our scale efficiency mechanism, with its relatively moderate score potentially reflecting uneven adoption of large-scale farming in the sample [32]. Collectively, these covariates reinforce that the OSCF policy operates through interconnected channels of technical advancement, structural adjustment, and regional development capacity.

### 4.6. Policy implications

Our findings offer several policy implications for promoting sustainable agricultural development. Firstly, the OSCF policy demonstrates the potential of agricultural subsidies to reduce chemical fertilizer use, crucial for long-term sustainability in China and globally. Policymakers should recognize and leverage the significant role of agricultural subsidies in fostering sustainability, potentially informing policy reforms in other regions.

Secondly, the technical effect of the OSCF policy highlights the importance of enhancing farmers’ access to advanced knowledge and environmentally-friendly agricultural techniques. Policymakers should prioritize measures to facilitate the adoption of new agricultural technologies through intensified agricultural technology dissemination, collaboration with enterprises and research institutions, and increased opportunities for farmers to participate in technical training and production experience sharing [26,113,120].

Thirdly, the scale effect of the OSCF policy emphasizes the benefits of larger-scale farming operations in achieving economies of scale and reducing fertilizer use. Policymakers should focus on strengthening land management and promoting rational land transfer to ensure efficient resource allocation and overcome the inefficiencies of small-scale farms. This includes providing incentives for the development of land rental markets and establishing more land transfer service platforms [1].

Finally, the heterogeneous analysis indicates that differentiated policy instruments are crucial for maximizing effectiveness of the OSCF policy across different regions. Policymakers should prioritize developed regions, cash crop production areas, and regions with high initial chemical fertilizer use to enhance the impact of the OSCF policy. For underdeveloped regions, grain-producing areas, and regions with low initial fertilizer use, additional support and complementary policy instruments should be considered to address specific challenges and promote sustainable agricultural practices.

### 4.7. Limitations and future works

This study offers a comprehensive understanding of the OSCF policy’s impact on chemical fertilizer use, providing valuable insights for policymakers and farm managers to promote sustainable agricultural development. However, certain limitations are acknowledged. Firstly, while many factors influence fertilizer use, this study focuses primarily on macro-level factors, potentially overlooking micro-level influences. Future research should integrate macro and micro data to consider all potential driving factors comprehensively. Secondly, although this study conducts heterogeneity analysis on policy effects and provide a brief illustration of key features, it has not fully examined the impact of all influencing factors on policy outcomes. Further investigation is needed to understand the contributions of different factors to fertilizer use reduction through subsidy policies. This will enable policymakers to implement more targeted measures and maximize policy efficiency. Besides, future research could integrate geospatial data to explore spatial autocorrelation or cluster effects. Such analyses would further elucidate how localized environmental and infrastructural factors moderate policy impacts. Lastly, this study only examines the policy impact within pilot areas, neglecting potential spillover effects on surrounding regions. Future research should consider the broader implications of agricultural subsidy policies to assess their full impact on sustainable agricultural development.

## 5. Conclusions

The excessive use of chemical fertilizers poses a significant challenge to the sustainable development of agriculture in China. In response, China has implemented various measures, including the OSCF policy, to reduce chemical fertilizer use. However, previous studies have either focused on evaluating the policy effects in specific regions or assessed the policy effects based on micro-level data, neglecting the potential heterogeneity of policy effects and the underlying mechanisms. This study employs DML approach to assess the impact and mechanisms of the OSCF policy on chemical fertilizer use, utilizing panel data from 2319 Chinese counties from 2012 to 2022. This study constructs an analytical framework to examine the direct and effects and underlying mechanisms of the OSCF policy, employing it as a quasi-natural experiment. Additionally, the research investigates the policy’s differential impacts across various regions, providing insights into the optimization of the OSCF policy for diverse regional settings. The results demonstrate that the OSCF policy effectively reduces chemical fertilizer use by improving technical and scale efficiency. Furthermore, the policy appears to be more effective in developed regions, cash crop production areas, and regions with high initial chemical fertilizer use.

## Supporting information

S1 File Estimation process of TE and SE. (PDF)

S1 Data Raw panel data for all indicators. (XLSX)

S2 Data Data for Fig 2. (XLSX)

S3 Data Data for Fig 3. (XLSX)
