# Supplementary material for: Impact of agricultural subsidy on chemical fertilizer use: Empirical evidence of China’s Organic-Substitute-Chemical-Fertilizer policy based on double machine learning
Source: PLoS One. 2025 Nov 5;20(11):e0334751. doi: 10.1371/journal.pone.0334751 (PMC12588528; doi:10.1371/journal.pone.0334751)
Supplement: S1 File — (PDF) [file pone.0334751.s001.pdf]

Estimation of TE and SE

Technical efficiency (TE) and scale efficiency (SE) are operationalized as mediating variables to quantify how the OSCF policy reduces chemical fertilizer use through efficiency improvements. TE reflects the efficiency of resource allocation (i.e., the extent to which a county achieves maximum output with given inputs), while SE measures the proximity to the optimal production scale (i.e., whether production operates at a scale where average costs are minimized). Both are estimated using an input-oriented Malmquist productivity index method, which focuses on minimizing input usage while maintaining a given level of output-consistent with the core goal of evaluating chemical fertilizer reduction effects.

The Malmquist productivity index, rooted in distance functions and non-parametric frontier analysis, is employed to measure total factor productivity (TFP) changes over time. In the context of this study, it is applied to decompose productivity changes into technical efficiency change (capturing improvements in resource utilization) and technical progress (which is not considered in this study because it is inadequate to assume that the implementation of OSCF policy can change the production frontiers). The decomposition further isolates scale efficiency (SE) and pure technical efficiency (TE), aligning with the input-oriented framework specified in Section 3.1.4.

The Malmquist index between periods *t* and *t*+1 is computed as the geometric mean of two indices, each relative to a different technology:

$$M(x^{t+1}, y^{t+1}, x^t, y^t) = \left[\frac{D^t(x^{t+1}, y^{t+1})}{D^t(x^t, y^t)} \times \frac{D^{t+1}(x^{t+1}, y^{t+1})}{D^{t+1}(x^t, y^t)}\right]^{1/2}$$

where *x* and *y* are the input and output respectively, *D* means the distance function.

The Malmquist index is decomposed into two components, i.e., technical efficiency change and technical progress. Technical efficiency change is further decomposed into pure technical efficiency change (which is defined as TE in this study) and scale efficiency change (which is defined as SE in this study) by comparing results under constant returns to scale (CRS) and variable returns to scale (VRS) assumptions:

$$TE = \frac{D_{VRS}^{t+1}(x^{t+1}, y^{t+1})}{D_{VRS}^{t}(x^t, y^t)}$$

$$SE = \frac{D_{CRS}^{t+1}(x^{t+1}, y^{t+1})/D_{VRS}^{t+1}(x^{t+1}, y^{t+1})}{D_{CRS}^{t}(x^t, y^t)/D_{VRS}^{t}(x^t, y^t)}$$

where D_{VRS} and D_{CRS} denote the distance function under VRS and CRS, respectively. In practice, the Malmquist index and its components are computed using Data Envelopment Analysis (DEA) to estimate the distance functions. For each period *t* and *t*+1, four DEA models are solved for each decision-making unit (DMU, i.e., each county in this study):

CRS model for period *t*:

$$D_{CRS}^{t}(x^t, y^t) = \min_{\theta,\lambda}\theta \text{ s.t. } \sum_{j=1}^{n} \lambda_j y_j \geq y^t,\ \sum_{j=1}^{n} \lambda_j x_j \geq \theta x^t,\ \lambda_j \geq 0$$

CRS model for period *t*+1:

$$D_{CRS}^{t}(x^{t+1}, y^{t+1}) = \min_{\theta,\lambda}\theta \text{ s.t. } \sum_{j=1}^{n} \lambda_j y_j \geq y^{t+1},\ \sum_{j=1}^{n} \lambda_j x_j \geq \theta x^{t+1},\ \lambda_j \geq 0$$

VRS model for period *t*:

Add the constraint $\sum_{j=1}^{n} \lambda_j = 1$ to the CRS model.

VRS model for period *t*+1:

Add the constraint $\sum_{j=1}^{n} \lambda_j = 1$ to the CRS model.
